# High prevalence of the *mcr-1* gene in retail chicken meat in the Netherlands in 2015

**DOI:** 10.1186/s13756-017-0242-8

**Published:** 2017-08-18

**Authors:** Eefje J. A. Schrauwen, Pepijn Huizinga, Nick van Spreuwel, Carlo Verhulst, Marjolein F. Q. Kluytmans-van den Bergh, Jan A. J. W. Kluytmans

**Affiliations:** 1 Laboratory for Microbiology and Infection Control, Amphia Hospital, Breda, The Netherlands; 2 Academy for Technology and Environmental Health, Avans University of Applied Sciences, Breda, the Netherlands; 3 Laboratory for Medical Microbiology and Immunology, Elisabeth-TweeSteden Hospital, Tilburg, the Netherlands; 4 Julius Centre for Health Sciences and Primary Care, University Medical Center Utrecht, Utrecht, The Netherlands; 5 Amphia Academy Infectious Disease Foundation, Amphia Hospital, Breda, The Netherlands; 6 Department of Medical Microbiology and Infection Control, VU University Medical Center, Amsterdam, The Netherlands

**Keywords:** Colistin resistance, Chicken meat, *mcr-1*, Netherlands, Prevalence, PCR method, Enterobacteriaceae

## Abstract

Recently, plasmid-mediated colistin resistance was reported in humans, animals and food. We studied the presence of *mcr-1* and *mcr-2* in Dutch retail chicken meat. The prevalence of *mcr-1* was 24,8% (53/214), whereas *mcr-2* was not found. The presence of *mcr-1*-positive Enterobacteriaceae was confirmed by culture in 34/53 samples (64,2%). The prevalence depended on the supermarket chain and was lower in free-range chicken samples. The unexpected high prevalence of *mcr-1* in food is cause for concern.

## Article

Recently, a plasmid-mediated colistin resistance gene, called *mcr-1*, was reported from China [1], which was soon followed by several reports on *mcr-1* positive Enterobacteriaceae from food, animals and the environment across the world [2, 3]. This is of particular concern as colistin is currently considered as a last resort agent for treatment of infections with isolates that contain other resistance traits, like extended-spectrum beta-lactamase (ESBL) producing Enterobacteriaceae or carbapenem-resistant bacteria [4–6].

Recent investigations using metagenomics, indicated a substantial larger environmental reservoir regarding the *mcr-1* gene in the Chinese population [7]. This indicates that other approaches are needed to reveal the true reservoir of *mcr-1*.

In the Netherlands, *mcr-1* was detected at low prevalence in *E. coli* isolates from livestock and meat (< 2%) and at very low frequencies in the human population [6, 8, 9]. The aim of this study was to determine the prevalence of *mcr-1* and *mcr-2* in a collection of poultry samples from Dutch supermarkets using a PCR-based method.

## Collection and analysis of retail chicken samples

Chicken meat samples (*n* = 214) were bought from four supermarket chains throughout the Netherlands in 2015. The number of samples was balanced across supermarkets and one sample per production batch was included. Meat samples were enriched overnight in non-selective tryptic soy broth (TSB) and subsequently stored at -80 °C until further testing. DNA was isolated from 50 μl of the defrosted TSB using NucliSens EasyMAG (Biomérieux). Detection of *mcr-1* and *mcr-2* gene was performed by real-time multiplex PCR (ABI 7500 system) using the following primers and probes: mcr1-2_forward AAATGCCMTRCARACCGACCAAG, *mcr-1*-2_reverse TCTCACCGACGACGAACACCAC, *mcr-1*_probe YY-BHQ1 TTTGATGCGCCGATTGGGCTTGATC, *mcr-2* probe FAM-BHQ1 TGCAGACCACCAAGCCGAGCGAG. Control isolates that contained either *mcr-1* or *mcr-2* were used. Concurrently, 100 μl of TSB was inoculated in fresh TSB and incubated at 35-37°C overnight. Subsequently, 10 μl of this overnight grown TSB was streaked onto a CLED-colistin-agar with 1.5 μg/ml colistin (Duchefa) and 10 μg/ml Daptomycin (Novartis). All colistin resistant isolates that could grow on the selective CLED-colistin-agar were confirmed by Vitek MS (Biomérieux) and non-intrinsic colistin resistant isolates found, were further tested for the presence of mcr genes by PCR. The isolates were tested by broth-micro-dilution (BMD), in cation-adjusted Mueller Hinton broth [10], for colistin susceptibility and Vitek2 (AST N344) (Biomérieux) to determine the susceptibility for various other antibiotics.

## Prevalence of *MCR-1* in retail chicken samples

The prevalence of *mcr-1* on retail chicken meat using PCR on TSB was 24.8% (53/214 samples positive) and no *mcr-2* was detected. Using a selective culture method, the presence of *mcr-1* was confirmed in 34 of these 53 (64.2%) samples with a positive result by PCR. Using this culture method, intrinsically resistant isolates had the ability to grow as well. No mcr-positive Enterobacteriaceae were found in all other samples. *E. coli* was identified in 32 samples, and *K. pneumoniae* in two samples. The median CT-value of the culture positive samples was significantly lower: culture-positive, 25.6 and culture-negative, 30.1, (*p* < 0.001, Mann-Whitney U-test).

**Fig. 1:**
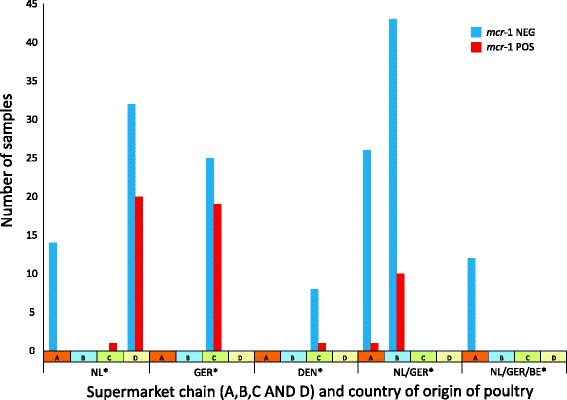
Distribution of the *mcr-1* positive and negative chicken meat samples across supermarket chains and country of origin (*n* = 214)

The prevalence of *mcr-1* according to the method of farming husbandry (free range: yes/no) and supermarket chain is shown in Table 1. Using multivariable regression analysis it was shown that both variables were statistically significant and independently related to the presence of *mcr-1*. We also investigated the country of origin (COO), as indicated on the label (Table 1). This variable showed co-linearity with the supermarket chain and was not included in the multivariate analysis (Fig. 1).

**Table 1:** Determinants of the presence of *mcr-1* in Dutch retail chicken meat samples, 2015

Determinant	Samples n = 214	mcr-1 PCR positive n (%)	OR (95% CI)	Adjusted OR (95%CI)
Labelling as free-range				
Yes	70	10 (14.3)	reference	reference
No	144	43 (29.8)	2.6 (1.2-5.5)	3.0 (1.3-6.6)
Supermarket chain				
A	53	1 (1.9)	reference	reference
B	53	10 (18.9)	12.1 (1.5-98.3)	12.5 (1.5-101.8)
C	54	21 (38.9)	33.1 (4.2-257.8)	34.6 (4.4-272.0)
D	54	21 (38.9)	33.1 (4.2-257.8)	37.5 (4.8-295.3)
Country of origin^a^				
NL	67	21 (30.3)	2.9 (1.3-6.5)	
GER	44	19 (43.2)	4.8 (2.0-11.4)	
DEN	9	1 (11.1)	0.8 (0.1-6.9)	
NL/GER	80	11 (13.8)	reference	
NL/GER/BE	12	0 (0.0)	not applicable	
Unknown	2	1 (50.0)	6.3 (0.4-107.8)	

^a^ *NL* The Netherlands, *GER* Germany, *DEN* Denmark, *BE* Belgium

The in vitro antimicrobial susceptibility for the 35 *mcr-1* positive isolates, which were found by culture, is shown in Table 2. One sample (nr. 11) harboured two isolates which were *mcr-1* positive but with different susceptibility patterns. There were high levels of resistance against ampicillin (100%), amoxicillin-clavulanic acid (89%), trimethoprim/sulfamethoxazol (69%) and ciprofloxacin (57%). Only one *mcr-1* positive ESBL-producer was found (sample 34) and all isolates were susceptible to meropenem.

**Table 2:**
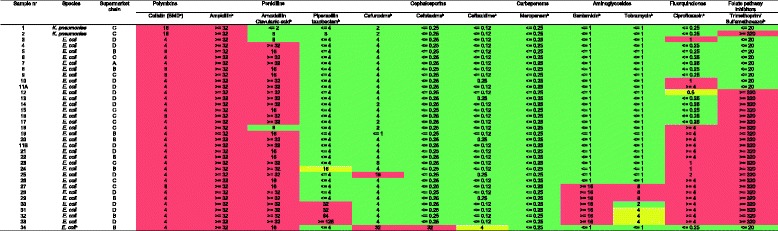
Antimicrobial susceptibility of *mcr*-1 positive Enterobacteriaceae isolated from Dutch retail chicken meat, 2015

^a^ *BMD* broth micro-dilution

^b^Vitek2

^c^Phenotypic confirmed extended-spectrum beta-lactamase- producing *E. coli*

Red in the table represents resistant, yellow intermediate and green suscpetible for the given antibiotics according to EUCAST guidelines

## Discussion

In this study, a PCR-based detection method identified an unexpected high prevalence (24.8%) of *mcr-1* in retail chicken meat samples and no *mcr-2* was found. The majority of the PCR positive samples were confirmed by selective culture. The 19 PCR positive samples that could not be confirmed by culture were all overgrown by intrinsically colistin-resistant bacterial species (e.g. *Serratia spp.* and *Proteus spp.*), which decreases the sensitivity of the culture for *mcr-1* harbouring bacteria. In addition, the higher CT-values in the culture-negative samples are indicative of a lower bacterial load, which may further explain the negative findings. Moreover, the freeze-thaw step of the samples might have played a role in the viability of the colistin-resistant bacteria.

A link between *mcr-1* in humans and food has been proposed in the first report from China, in which 28% of poultry samples harboured *mcr-1* [1]. In a study from south America, chicken meat was also identified as a reservoir for *mcr-1*-harboring *E.coli* isolates (19.5%) based on a selective culture approach. It should be realized that Brazil is the third-largest chicken meat producer and the largest exporter of this product [11]. Subsequent studies confirmed the presence of *mcr-1* in isolates from poultry and other meat products from Europe, but at much lower rates [2]. A recent study form Germany, including 580 *E. coli* isolates from chicken meat, found a decreasing prevalence of *mcr-1*, from 8.1% in 2011 to 0.5% in 2014, however, this was based on isolate screening [12].

At present, *mcr-1* is only sporadically found in humans in the Netherlands [6, 8, 9]. This is in a situation where colistin and other polymyxins are used at very low levels. In 2014, polymyxins constituted less than 0.1% (0.01 defined daily dose (DDD)/1000 inhabitant-days) of all systemic antimicrobials used in primary care and 0.3% (0.2 DDD/100 patient-days) in the hospital setting [13]. Therefore, the selective pressure is currently low. Also, it should be taken into account that more selective approaches are necessary to reveal the true presence of *mcr-1* in humans. Both the current study and the study by Wang et al. show that direct molecular techniques, molecular techniques after enrichment steps and selective culture techniques result in much higher prevalences compared to studies using non-targeted methods [7]. Considering the low selective pressure in humans and the lack of data on the resistome in humans it is not evident what the implications of these findings are for public health on the short or long term.

The culture approach showed that the majority of the *mcr-1* positive isolates were susceptible to cephalosporins, carbapenems and aminoglycosides. Apparently, the *mcr-1* gene is frequently present in isolates that are susceptible to most classes of antibiotics. This might explain the relative low prevalence of *mcr-1* in studies that have primarily focused on isolates with other resistance traits [6, 14–17].

The differences in *mcr-1* prevalence between supermarket chains are remarkable, with the two chains with the highest prevalence (C and D) having an odds ratio that is approximately 35 times higher compared to the supermarket chain with the lowest prevalence, after adjusting for free-range rearing of the animals. We attempted to extend the multivariable regression analysis to study the reservoir of the *mcr-1* gene to COO. However, in most cases, multiple countries are named on one sample without further specification. In addition, there was co-linearity with the supermarket chain (Fig. 1), prohibiting to include these variables in the regression model. Further details on the production process could not be studied as the label on the package does not provide further information. The conclusion is that there are large differences in the prevalence of *mcr*-1 between supermarkets which we cannot explain with the available information. As shown in Table 2, there were variable susceptibility patterns to other antibiotics, which showed a tendency to cluster within supermarket chains. We cannot draw conclusions based on these data. This would require additional research. It would be important to extend the investigations into the different chains of production of chicken meat to identify the determinants of the presence of *mcr*-1.

In conclusion, we have shown a high prevalence of *mcr-1* in chicken meat with a large and unexplained variation between supermarket chains. The approach, specifically targeting the presence of *mcr-1*, resulted in a much higher prevalence than previous studies that did not specifically target colistin resistance. These findings warrant further studies to elucidate the underlying mechanisms of spread and the genetic location of the *mcr*-1 gene. Moreover, continued monitoring of the potential reservoirs for this plasmid-mediated colistin resistance is of utmost importance.

## Abbreviations

COO: Country of origin
DDD: Defined daily dose
ESBL: Extended-spectrum beta-lactamase
